# Supplementary figures and images for: Junior scientists spotlight social bonds in seminars for diversity, equity, and inclusion in STEM
Source: PLoS One. 2023 Nov 2;18(11):e0293322. doi: 10.1371/journal.pone.0293322 (PMC10621980; doi:10.1371/journal.pone.0293322)

**a**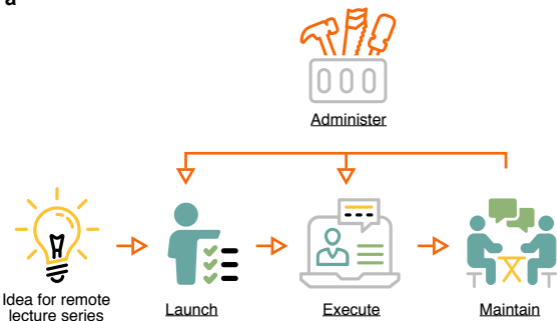**b**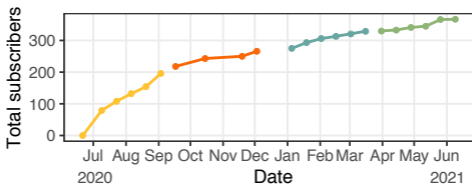**c**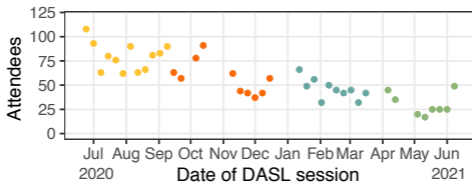

Supplement: S1 Fig — a) Outline of running a remote lecture series. We provide guides detailing how to approach each underlined component on our website. b) Total subscribers to the DASL mailing list over time. c) The number of attendees per DASL session over the first year of programming. (PDF) [file pone.0293322.s001.pdf]

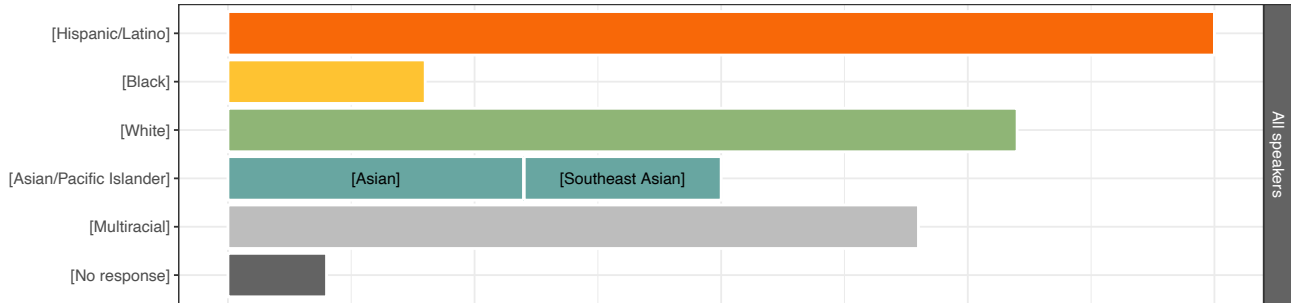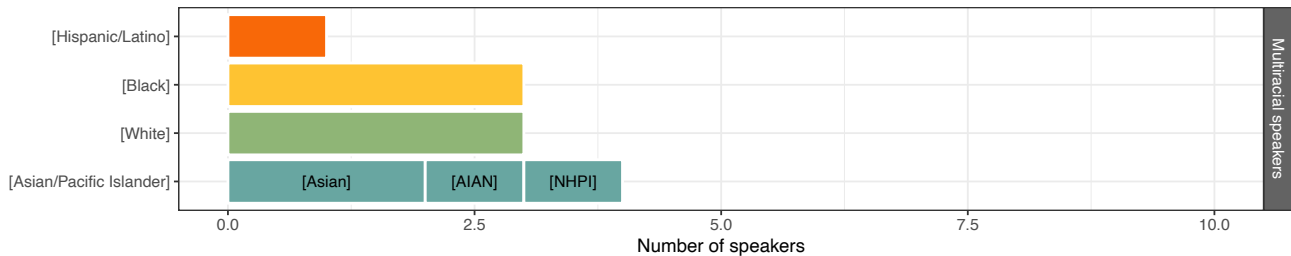

Supplement: S3 Fig — Counts of racial identities separated by speakers who identify as a single race or multiracial. Labels were taken directly from the Speakers page of the DASL website. (PDF) [file pone.0293322.s003.pdf]

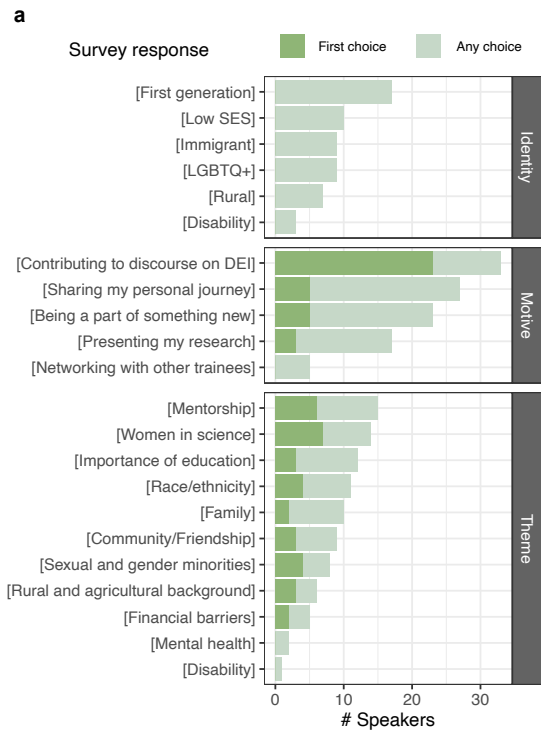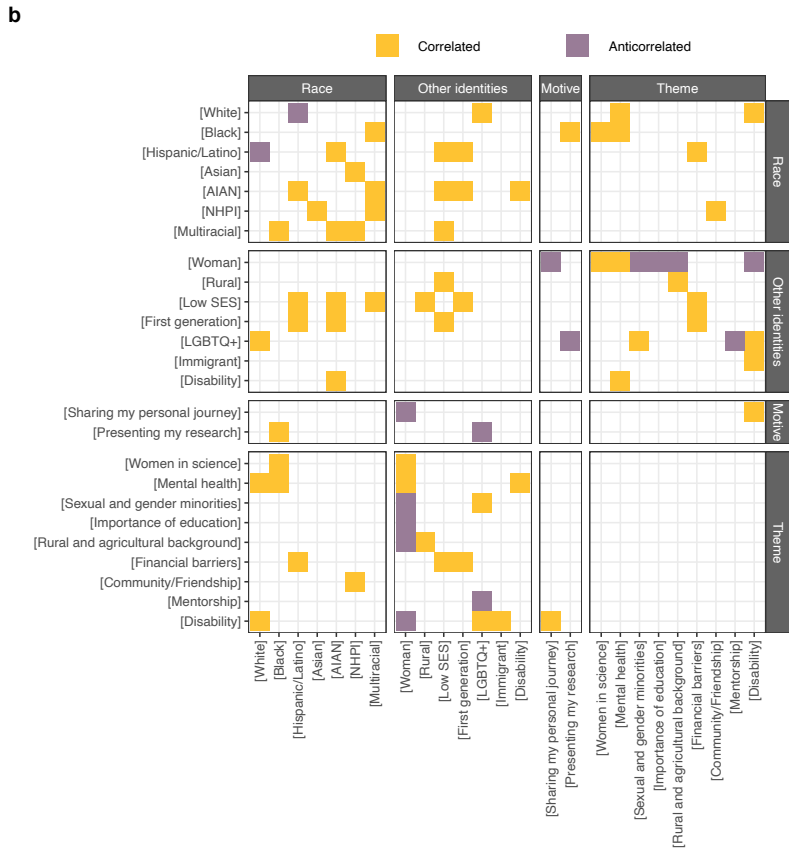

Supplement: S4 Fig — a) Counts of speakers who selected each identity, interest, and talk theme. The top ranking talk theme selected by the speaker is indicated in a darker shade of green. b) Correlated and anticorrelated identities and talk themes for DASL speakers who posted data to the DASL website. (PDF) [file pone.0293322.s004.pdf]
