# Supplementary material for: Junior scientists spotlight social bonds in seminars for diversity, equity, and inclusion in STEM
Source: PLoS One. 2023 Nov 2;18(11):e0293322. doi: 10.1371/journal.pone.0293322 (PMC10621980; doi:10.1371/journal.pone.0293322)
Supplement: S2 Fig — Speaker name associations from the Fragile Nucleosome seminar series. Counts are tallied as in Fig 3A. (PDF) [file pone.0293322.s002.pdf]

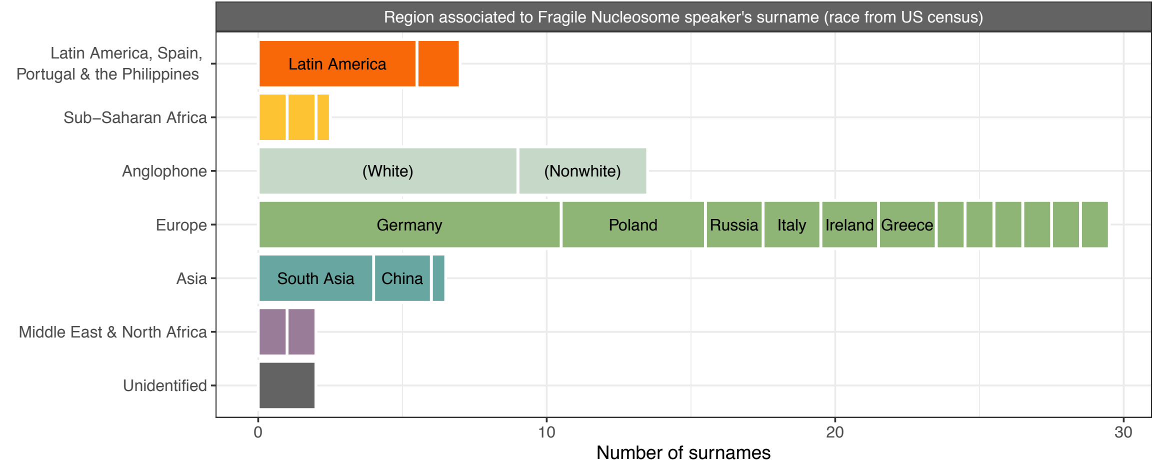
Region associated to Fragile Nucleosome speaker's surname (race from US census)
Latin America, Spain, Portugal & the Philippines
Sub−Saharan Africa
Anglophone
Europe
Asia
Middle East & North Africa
Unidentified
Latin America
(White)
(Nonwhite)
Germany
Poland
Russia
Italy
Ireland
Greece
South Asia
China
0
10
20
30
Number of surnames
