# Supplementary material for: Junior scientists spotlight social bonds in seminars for diversity, equity, and inclusion in STEM
Source: PLoS One. 2023 Nov 2;18(11):e0293322. doi: 10.1371/journal.pone.0293322 (PMC10621980; doi:10.1371/journal.pone.0293322)
Supplement: Attachment — Submitted filename: Response to Reviewer Round 2.docx [file pone.0293322.s007.docx]

Reviewer #1: The authors have largely addressed my concerns, except I still find the motivation around name-based region identification lacking.

We appreciate the reviewer’s contributions to this work and are happy to respond to the remaining concerns below.

The authors have added a self-report survey for validating the name-based tool, which is commendable and strengthens the analysis. However, I still don't understand why they use name-based tools rather than just looking up each of the speakers and manually guessing the region themselves. Is it because it is too hard to guess regions from surnames? Or is it because of discomfort around classifying people demographically by hand?

I find the former reason more defensible than the latter. What I want to prevent is a situation where an algorithmic tool is used to bypass or substitute an ethical consideration. For example, if the authors consider it too problematic to label people by hand, then they shouldn't use an algorithm to do it either! Or rather, if both are fine (which I think it is), and if humans are more accurate, then humans should just do it instead of kicking it off to an algorithm.

We believe that the introduction succinctly summarizes the benefits of computational audits of diversity:

Programmatic and semi-automated methods that make inferences based on names or documents have the potential to curtail response bias, protect participant privacy (e.g., no exposure of phone number, mailing address, or email address), and rescue data lacking self-identification. Indeed, participant name is sufficient for scalable and reproducible (if uneven) inference of race, nationality, gender, and geographic associations [[8–12]](https://paperpile.com/c/SP2vTd/Py8Yg+iAQsT+F8npo+BOm9a).

To reiterate our findings we added two sentences to the Discussion:

Our semi-automated approach based on the Forebears geospatial names database eliminated errors and irreproducibility due to subjective classification of speaker backgrounds and simplified comparisons across platforms. We also note that the Forebears API can process thousands of surnames near instantaneously with reduced data entry burden.

Having executed this work, the alternative of performing web searches of over 100 individuals and attempting to manually assess their geographic, racial, or ethnic diversity is not an attractive option even if comparable accuracy were attainable. In other settings, such as conferences with thousands of people, it would be prohibitive.

There may be additional information that can be gleaned from a Google search, but in our experience, geographic associations at the granularity presented (e.g. Middle East and North Africa, Hispanic/Latino, or Southeast Asia background) is usually not discernible for junior researchers if databases akin to Forebears are omitted. We are confident that the 4 billion records housed in the Forebears database trumps even expert familiarity with name origins if one were to guess without assistance.

We believe undocumented reasoning and irreproducible results are by far the greatest ethical peril this type of work presents. If subjective judgments were the foundation of the conclusions, the results could easily be skewed (consciously or unconsciously). Our approach is algorithmic, but it is simple to implement and understand. Even if several pages of documentation were included to justify every piece of evidence and analysis in a manual review (e.g. from examining pictures of speakers), future studies or replications would likely differ at least modestly in terms of classification bias.

Minor comments:
- P8: supplemental figures are referred to like (S1a Fig) and (S1b Fig), while later in P9, it is (S Fig 2). Make labeling scheme consistent. I prefer a scheme like "Fig 1" for main text and "Fig S1" for supplement, so that it is consistent that "Fig" comes first.

Good catch. We missed the callout to S Fig 2 when renaming figures to abide by PLOS One guidelines, which declare that Supporting information items be named S[i] Fig or S[i] Table.

- P16: the dashes surrounding "especially Asian men speakers" should be em-dashes

Another good find. We replaced hyphens and en-dashes with minus signs and em-dashes as needed here and elsewhere throughout the manuscript.

Reviewer #2: I thank the authors for their efforts to improve the manuscript, especially in addressing my concerns regarding the narrative and implications. I believe that the new version of the publication is much stronger and should be published in PLoS One pending only a few exceedingly minor edits.

We’re grateful to the reviewer for offering such detailed feedback in the previous round. We are further enthused by the encouraging remarks on the current draft.

Minor comments:
- I notice that the authors never actually define the acronym DEI. It is widely used, but it would still be good practice to spell out the full phrase when first used in the manuscript.

This was indeed an oversight. The acronym DEI is now specified in the first sentence of the Introduction:

Following spring of 2020, broader recognition of widespread social injustice spurred advocacy for diversity, equity, and inclusion (DEI) in STEM.

- In a similar vein, the authors use the phrase “equity, diversity, and inclusion” in several spots, but also use the acronym DEI. For the sake of consistency, it would be good to always use a consistent ordering, or to always use the acronym.

We agree entirely. All ordering is consistent with “diversity, equity, and inclusion” and most references to diversity, equity, and inclusion are referred to with “DEI” to be more consistent.

- Page 3: I appreciate the new methodological paragraph in the introduction that outlines the benefits and drawbacks of programatic inference of speaker identify. However, I feel that it is missing a thesis statement that connects it cleanly with the narrative of the introduction. Perhaps the author could add one sentence in the form: “For these reasons, we choose to use programmatic inference of identify based on each speaker’s name, rather than relying on self-report information.

We agree that the previous draft did not explicitly connect the listed concerns for automated methods for assessing diversity to the methodology we undertook. We added a sentence to the last paragraph of the Introduction that expresses the sentiment requested: namely that programmatic inference produces a greater volume of usable data that is more reproducible.

Here, we summarize how trainee life scientists conceive of DEI in STEM (Fig 1b). We aggregated gender identity from speaker presentations, algorithmically inferred geography from speaker surnames, and topic mentions gleaned from seminar transcripts to produce highly powered reproducible datasets not limited in scope by preconceived survey questions or speaker response rate in post hoc self-reported information.

- Page 5: When mentioning packages like tidy text to SnowballC, the authors should also specify that these are packages in R.

The methods section now clearly identifies the R packages used.

Terms in talk titles were counted by parsing titles posted on the DASL website using the R packages tidytext and SnowballC.

Surnames assigned to the Anglophone region were further annotated with race using the predictrace::predict_race command from the predictrace R package.

Binomial confidence intervals were obtained using the binconf command from the Hmisc R package.

- Page 6: The authors reference a GitHub repository that contains the Wiki2019-LSTM model. It might make sense to also reference the publication with which the repository is associated: <https://www.cell.com/cell-systems/pdf/S2405-4712(21)00285-4.pdf>

We appreciate the reviewer pointing this out. The publication is cited in the results section, but now that the methods section is listed before the results section we believe it is essential to cite it there. It is now called out in both places.

We compared our geospatial name inferences to output from a recently published long short-term memory model called Wiki2019-LSTM [[8]](https://paperpile.com/c/RDPeYr/sq1O). The Wiki2019-LSTM model was downloaded from its GitHub repository (<https://github.com/greenelab/wiki-nationality-estimate>).

- Page 7: The authors state that manuscripts were uploaded to YouTube to obtain transcripts. To clarify, does that mean that the transcripts are generated using YouTubes’s automatic transcription software? If so, I would make that more explicit in the text, as well as briefly discuss the quality of transcriptions and its limitations.

That is correct: the transcripts are automatically generated by YouTube after uploading the recording. Anecdotally we heard from multiple sources that YouTube’s transcripts were the highest quality free or low-cost commercial option (i.e. without manual review by professionals). We found a reference from Consumer Reports that benchmarked various services and concluded the same.

Publicly viewable DASL and SQUAD talks were downloaded and then uploaded to YouTube, which automatically generated English language captions. Downloaded captions served as talk transcripts. We observed higher fidelity using YouTube than competing automated services such as Zoom, consistent with reports that YouTube produces nearly half as many errors in its audio transcripts: 5 words out of 100 for YouTube versus 8 for Zoom [[17]](https://paperpile.com/c/RDPeYr/sbJH). Transcripts were reviewed and keywords tallied in R.
